# Adolescents’ Self-Esteem in Single and Two-Parent Families

**Published:** 2014-04

**Authors:** Ali Alami, Shahla Khosravan, Leila Sadegh Moghadam, Fateme Pakravan, Fateme Hosseni

**Affiliations:** 1 Social Determents of Health Research Centre, Department of Public Health, School of Health, Gonabad University of Medical Sciences, Gonabad, Iran;; 2 Social Determents of Health Research Centre, Department of Community and Mental Health Nursing, School of Nursing and Midwifery, Gonabad University of Medical Sciences, Gonabad, Iran;; 3 Department of Community and Mental Health Nursing, School of Nursing and Midwifery, Gonabad University of Medical Sciences, Gonabad, Iran;; 4 Student Research Committee, Gonabad university of Medical Sciences, Gonabad, Iran

**Keywords:** Adolescence, Parenting Behavior, Nuclear Family, Self-Esteem, Single-Parent Family

## Abstract

**Background: **Self-esteem is one of the basic needs for all individuals especially in adolescence. The aim of this study was to determine associations between adolescents’ self-esteem and perceived maternal parenting styles as well as its dimensions in terms of family type.

**Methods: **In this analytic cross-sectional study, 356 high school students (250 two-parent nuclear family and 106 single-parent family) participated and filled out the Coppersmith self-esteem and the Robinson and colleagues (2001) perceived parenting styles questionnaires. Data were analyzed by SPSS software, version 18. To assess the relationship between participants’ self-esteem and parenting styles and dimensions, Mantel–Haenszel Chi-square test was used to adjust the effect of potential confounder variables. P≤0.05 was considered statistically significant.

**Results: **From a total of 370 questionnaires, 356 questionnaires were completed. The mean±SD of the participants’ self-esteem score was 38.49±6.55. Mean±SD of self-esteem score among the two-parent and single-parent students was 39.06±6.36 and 37.42±7.28, respectively (P=0.034). Dominant parenting style in both families was authoritative style. There were significant associations between the respondents’ self-esteem and their perceived parenting styles, after matching sex, family income, level of education, and parents job (P<0.005).

**Conclusion: **The results of this research can be used in educational interventions to modify the dimensions of parenting styles and improve self-esteem. Therefore, considering the relationship between child-rearing style and adolescent self-esteem, assessing other relating factors with adolescent self-esteem especially in single-parent family, such as father absence stigma, is suggested.

## Introduction

Self-esteem, defined as the general self-evaluation of a person toward himself/herself,^1^ is one of the main human needs; it is actually a characteristic of a normal person.^1^^,^^2^ Many psychologists know its deficiency as the root of many psychological diseases.^3^^-^^5^ This characteristic is more important in adolescence, because adolescence is considered as one of the main and dominant processes of social and psychological growth of personality.^6^ Although many factors have been reported in relation to self-esteem such as specific language impairment,^7^ child personality characteristics, weight status, socio-demographics, hispanic race, gender,^8^ and family structure^9^^,^^10^ but the relationship between self-esteem, parents’ attachment and parenting style has been emphasized in other studies.^3^^,^^11^^,^^12^

As noted, family structure and their emotional function are also among the important social aspects for development of self-esteem. Father and mother are the two main bases of a family. Changes in family structure and the absence of a parent in a family environment disrupt its balance and put the children in unfavorable and undesired conditions as compared to the children of two-parent families as it may have adverse effects on their normal growth.^13^ Several researchers have evaluated the health status of children in single-parent families^14^ and also parenting style and its relationship with behavioral and emotional characteristics of children such as sense of loneliness^15^ and self-esteem of children especially in families with two-parents or a single parent regardless of the cause of having single parent family and often focusing on divorce or being parent without marriage. They have suggested that there is a positive relationship between parenting styles, especially authoritative and indulgent parenting style, and increased self-esteem.^14^ Also, Abel et al. (1996) found 4 types of parenting strategies in a low-income sample of African American lonely mothers including (1) emotionally democratic and supportive, (2) low emotional involvement, (3) high parent–child conflict, and (4) high emphasis on emotional and behavioral control.^16^

Parenting styles are categorized differently. aAccording to demanding dimension, control dimension and also accepting and responsive dimension, Baumirind (1991) categorized the parenting style into three areas of logical authority (high in demand and control, high in accepting and responsive), authoritarian (high in demanding and directive, low in responsive), and permissive (low in demand and control,  high in accepting and responsive).^17^ Some studies reported that parenting style and its effects depend on cultural conditions^18^ and even specific body conditions such as congenital heart disease.^19^

However, it seems that few studies have evaluated the status of adolescents’ self-esteem and perceived parenting style and its dimensions especially from the adolescents’ perspective despite its more importance in orphan adolescents in comparison with adolescents in two-parent nuclear families. Therefore, this study was performed to determine and compare the adolescents’ self-esteem and perceived parenting styles and its dimensions and also to determine the relationship between self-esteem and perceived parenting style between two groups of adolescents: orphan single-parent families supported by a widow mother and two-parent nuclear families. The findings probably increase the nurses’ knowledge about these families as susceptible clients of nurses, especially community health nurses,   

## Materials and Methods

This was an analytic, cross-sectional study conducted on high school students in Gonabad, one of the eastern cities of Iran in 2012. Based on the previous studies,^20^ the required sample size was estimated at 265. [N=(z^ 2 ^σ^ 2 ^)/(d^ 2 ^); Z=1.96; σ=8.30; d=1]

We selected the sample from 15 to 18 year old high school students, who seemed healthy and had no physical and psychosocial problem according to self and family reports. Also because of the importance of adolescents’ parent status in terms of two-parent or single-parent and their potential different impacts on adolescents’ self-esteem, the sampling was performed in the two groups of adolescents with two-parents and with single-parent supervised by mother due to father’s death, for a minimum of one year. Using family health records available in three health centers of Gonabad, students with two-parent (n=265) and single-parent (n=106) were selected via systematic sampling and census, respectively. So, we finally selected 371 students in single-parent families supported by a widow mother and two-parent nuclear families. Those who were not willing to participate or did not complete the questioners were excluded from this study. The study was approved by the Ethics Committee of Gonabad University of Medical Sciences. Researchers initially explained the objectives of the study for the parents and obtained written informed consent for participation of their adolescents in the study. We then distributed the questionnaires among the adolescents after obtaining their verbal consent. Finally, the questionnaires were collected immediately upon completion. 

To collect data, we applied a three-section questionnaire including demographic factors, information on the status of self-esteem, and perceived maternal parenting styles from the adolescents’ point of view. Using the first part of the questionnaire, we collected the participants’ demographic characteristics such as gender, level of education and job of the parents, parental status, and family income. To assess the students’ self-esteem, we used Coopersmith self-esteem questionnaire (1976) (form A), as the second part of the questionnaire. Cooper Smith has reported a reliability coefficient of 0.88 after 5-weeks of test-retest and 0.7 after 3 years for the questionnaire.^21^ Purshafei, using split-half method, reported its reliability as 83.0 and Golbargi using test-retest method reported its reliability as 8.0. Form A of this questionnaire includes 85 items, 8 items of which are lie-evaluation. The mode of scoring of this test is zero and one. The minimum score that a person can obtain is zero and maximum score is 50. People who are scored above 25 are considered as high self-esteem and those who obtained a score less than 25 are considered as low self-esteem.^8^

In the third part of the questionnaire, to identify perceived maternal parenting style and its dimensions by adolescents, Robinson et al. used perceived maternal parenting style questionnaire which determins 3 styles of authoritative, authoritarian, and permissive and has 7 relational dimensions in addition to 3 parenting styles. The questionnaire includes 32 questions which is scored in Leakert scale as 1=never, 2=sometimes, 3=moderate, 4=most times, 5=always. Reliability and validity of parenting styles and its dimensions have been determined by Robinson et al., based on a sample of n=1377. These researchers have used Cronbach’s alpha coefficient and reported the reliability for authority dimension as 0.86, for authoritarian dimension as 82.0, and for permissive dimension as 64.0. Its validity was evaluated by factor analysis.^22^

This questionnaire was also used in Iran by Khoynezhad et al.^15^ In their study based on the sample group, consisting of 384 adolescent girls, the Persian version of this questionnaire was reported as valid and reliable. Cronbach’s alpha coefficient for authority, authoritarian, and permissive dimensions was reported 0.6, 0.83, and 0.61, respectively and the total alpha coefficient was 0.81. In Persian translation instead of the word “I” or “my wife”, the phrase “My Mother” is used and its validity using content validity has been reviewed and approved by experts. Since evaluation of authoritative, authoritarian and permissive parenting styles was performed with total scores of regulation, autonomy, physical coercion, verbal hostility, non-reasoning and indulgent dimensions, respectively, alpha coefficient of parenting style questionnaire for authoritative, authoritarian, and permissive styles were 0.61, 0.83, and 0.90, respectively and alpha coefficient of the total questionnaire was 0.81.^15^

In this study to determine the dominant perceived maternal parenting style, the scores obtained from questionnaire were standardized by (a/x)*100 where α is the score obtained from the questionnaire in each style and *Х* is the maximum score of that style. After standardizing the scores, each style which had the highest score was determined as perceived maternal parenting style from the students’ point of view.

Data analysis was performed using SPSS software, version 18. To assess the relationship between the participants’ self-esteem and parenting styles and dimensions, Mantel–Haenszel Chi-square test was used to adjust the effect of potential confounder variables. P≤0.05 was considered statistically significant. 

## Results

From a total of 370 questionnaires, 356 were returned. The data related to these questionnaires were used for the analysis (response rate=96%). In this study, mean±SD of the participants’ self-esteem score was 38.49±6.55. Table 1 shows demographic and family characteristics of participants and also the status of self-esteem distribution among them. In our study, according to the results of *t* test, mean scores of self-esteem among adolescents who lived in two-parent family (37.40±7.27) was significantly higher than those who lived in single-parent family (39.06±6.35) (P=0.034).

**Table 1:** Demographic and family characteristics of participants and self-esteem distribution among them

**Variable name**	**Number**	**Percentage**
Sex		
Female	169	48%
Male	187	52%
Family income		
Sufficient	267	75%
Insufficient	87	25%
Family situation		
Single-parent	106	30%
Two-parent	250	70%
Father education		
Low (below diploma)	197	57%
High (diploma and over)	150	43%
Mother education		
Low (below diploma)	253	72%
High (diploma and higher )	101	28%
Father job		
Governmental	97	28%
Nongovernmental	244	72%
Mother job		
Housewife	306	86%
Nonhousewife	48	14%
Self esteem		
Low	15	4%
High	341	96%

Perceived maternal parenting style and its dimensions in adolescents are presented in table 2.

**Table 2:** Perceived maternal parenting style and its dimensions in adolescents

**Parenting styles**	**Dimensions**	**mean±SD **
Authoritative		52.85±10.43
	Connection	17.17 ±3.88
	Regulation	17.61 ±4.11
	Autonomy	18.07 ±4.34
Authoritarian		21.78±7.05
	Physical coercion	5.67 ±2.35
	Verbal hostility	9.22 ±3.78
	Non reasoning/punitive	6.88 ±2.66
Permissive	Indulgent	10.27±3.43

The results showed that the percentage of authoritative parenting style was 90.7%, authoritarian 3.7%, and permissive 5.6%. table 3 shows the relationship between adolescents’ self-esteem and parenting styles and dimensions with control of possible confounding variables. As seen in table 3, the relationship between self-esteem and parenting style was significant after controlling the effects of gender, family income, education level and occupation of parents.

**Table 3:** The relationship between self-esteem and parenting style after controlling the confounding variables

**Variable name**	**Parenting style** **1=Authoritative** **2=Non authoritative**	**Self esteem**		P value*	**Test of homogeneity ** **(Breslow-Day)**	** OR_MH_** **(95% CI)**	**P value**
		**Low**	**High**				
		**Number (%)**					
Sex							
Female	1	5 (3.2%)	150 (96.8%)	0.105	0.865	0.178 (0.057-0.560)	0.005
	2	2 (14.3%)	12 (85.7%)				
Male	1	5 (3.0%)	163 (97.0%)	0.036			
	2	3 (15.8%)	16 (84.2%)				
Family income							
Sufficient	1	7 (2.9%)	238 (97.1%)	0.164	0.353	0.177 (0.056-0.560)	0.006
	2	2 (9.1%)	20 (90.9%)				
Insufficient	1	3 (3.9%)	74 (96.1%)	0.019			
	2	3 (30.0%)	7 (70.0%)				
Father education							
Low	1	7 (3.9%)	172 (96.1%)	0.542	0.109	0.233 (0.069-0.784)	0.038
	2	1 (5.6%)	17 (94.4%)				
High	1	3 (2.2%)	133 (97.8%)	0.011			
	2	3 (21.4%)	11 (78.6%)				
Mother education							
Low	1	7 (3.1%)	220 (96.9%)	0.071	0.378	0.178 (0.057-0.557)	0.004
	2	3 (11.5%)	23 (88.5%)				
High	1	3 (3.2%)	91 (96.8%)	0.038			
	2	2 (28.6%)	5 (71.4%)				
Father job							
Governmental	1	2 (2.2%)	89 (97.8%)	0.880	0.428	0.235 (0.068-0.809)	0.047
	2	0 (0.0%)	6 (100.0%)				
Nongovernmental	1	8 (3.7%)	211 (96.3%)	0.024			
	2	4 (16.0%)	21 (84.0%)				
Mother job							
Housewife	1	8 (2.9%)	267 (97.1%)	0.024	0.364	0.170 (0.054-0.538)	0.003
	2	4 (12.9%)	27 (87.1%)				
Nonhousewife	1	2 (4.3%)	44 (95.7%)	0.122			
	2	1 (50.0%)	1 (50.0%)				

*Fisher’s Exact Test

## Discussion

This study was performed to determine the adolescents’ self-esteem and perceived maternal parenting styles and its dimensions and also the relationship between the two groups of adolescents: orphan single-parent families supported by a widow mother and two-parent nuclear families. The results showed that mean score of adolescents’ self-esteem in widow-single-parent families was lower than that of the two-parent nuclear families and this difference was statistically significant. Despite our results and those of some other studies,^23^ Casey’s study showed that there was no relationship between child self-esteem and father’s presence.^24^ Coleman suggested the stigma of being a member of father absent households can be destructive, but it depends on a child’s development; also, if father’s absence is a norm in community, the stigma does not have the same impact on families who live in the communities where father absence is abnormal.^25^ In Iran, the most common type of household is the nuclear family; more than 80 percent of single parent families are formed due to the death of the father; others are due to divorce and/or mother’s death. Iranian society is a patriarchal society and family confers social status through a man; widowhood and father absence are stigmas. Such a family is named “bee sarparast” that means withouht a man supporter.^26^ Therefore, according to Coleman, lower adolescents’ self-esteem in Iranian widow-single-parent families is expected.^25^

The dominant parenting style was authoritative style from the viewpoint of both groups of adolescents in orphan single-parent families supervised by mother and adolescents in two-parent nuclear families. In a qualitative study on widows who were headof the family, the parenting style of these mothers was reported as sentimental with three characteristics: child centre, sacrifice and self-ignorance;^26^ this parenting style with a high level of support and low levels of control was similar to Baumrind’s indulgent-parenting and emotionally democratic and supportive parenting typology of Abell et al.^16^ This difference may be related to different understanding of mothers and adolescents from perceived parenting style.

There was a significant positive relationship between authoritative parenting style and adolescent self-esteem in both groups of two-parent and single-parent families headed by widowed mother and a significant negative relationship was observed between self-esteem and authoritarian style in both groups. Although these findings were not in accordance with the study of Gecas and Schwalbe which had suggested the cultural background,^27^ they were similar to many other studies (e.g. 9).

In this study, although researchers controlled the confounding factors such as gender, occupation and education level of parents, parents’ economic sufficiency and status that were reported as factors influencing self-esteem in some studies, a significant relationship was shown between self-esteem and democratic parenting style. This indicates the importance of the effect of democratic parenting style on adolescents’ self-esteem despite the other related factors.

Also, in this study there was a relationship between all dimensions of authoritarian parenting style (the physical coercion, verbal hostility, non-reasoning) and self-esteem and also between dimension of permissive parenting style (indulgent) and self-esteem in both groups; the dominant parenting style in both single-parent and two-parent groups was autonomy of the authoritative parenting style, but connection and regulation dimensions and self-esteem in single-parent group did not show a significant relationship while all dimensions of authoritative parenting style had a significant positive correlation with self-esteem in adolescence of nuclear families. This can be considered as a possible factor of differences in adolescents’ self-esteem in nuclear families and in single parent families in this study. In other studies, these components have shown a negative correlation with sense of loneliness.^15^

The limitation of this study was non-simultaneous evaluation of parenting styles from the viewpoint of adolescents and their parents. Also it was a correlation study; therefore, we could not find out the cause of differences in adolescents’ self-esteem in nuclear families and in single parent families in this study.  

## Conclusion

In this study, adolescents’ self-esteem in single-parent families was lower than that in the two-parent nuclear families. Although the dominant parenting style in both single-parent and two-parent groups was authoritative parenting style, there are differences between the two groups in terms of dimensions of parenting style. So teaching parenting style with emphasis on its dimensions to mothers of single-parent families is recommended to improve self-esteem in their children through providing interactive communication and flexible environment for observing the outcomes of parent’s behavior by children. Also, considering the relationship between child-rearing style and adolescent self-esteem, assessing other influential factors with adolescent self-esteem especially in single-parent family, such as father absence stigma, is suggested. 
